# The contribution of hypertensive disorders of pregnancy to late preterm and term admissions to neonatal units in the UK 2012–2020 and opportunities to avoid admission: A population‐based study using the National Neonatal Research Database

**DOI:** 10.1111/1471-0528.17574

**Published:** 2023-06-19

**Authors:** Frances Conti‐Ramsden, Jessica Fleminger, Julia Lanoue, Lucy C. Chappell, Cheryl Battersby, the UK Neonatal Collaborative

**Affiliations:** ^1^ Department of Women and Children's Health King's College London London UK; ^2^ Neonatal Medicine, School of Public Health, Faculty of Medicine Imperial College London London UK

**Keywords:** hypertension, hypoglycaemia, neonatal units, pregnancy

## Abstract

**Objective:**

To quantify maternal hypertensive disorder of pregnancy (HDP) prevalence in late preterm and term infants admitted to neonatal units (NNU) and assess opportunities to avoid admissions.

**Design:**

A retrospective population‐based study using the National Neonatal Research Database.

**Setting:**

England and Wales.

**Population:**

Infants born ≥34 weeks’ gestation admitted to NNU between 2012 and 2020.

**Methods:**

Outcomes in HDP infants are compared with non‐HDP infants using regression models.

**Main outcome measures:**

Hypertensive disorder of pregnancy, primary reason for admission, clinical diagnoses and resource use.

**Results:**

16 059/136 220 (11.8%) of late preterm (34^+0^ to 36^+6^ weeks’ gestation) and 14 885/284 646 (5.2%) of term (≥37 weeks’ gestation) admitted infants were exposed to maternal HDP. The most common primary reasons for HDP infant admission were respiratory disease (28.3%), prematurity (22.7%) and hypoglycaemia (16.4%). HDP infants were more likely to be admitted with primary hypoglycaemia than were non‐HDP infants (odds ratio [OR] 2.1, 95% confidence interval [CI] 2.0–2.2, *P* < 0.0001). 64.5% of HDP infants received i.v. dextrose. 35.7% received mechanical or non‐invasive ventilation. 8260/30 944 (26.7%) of HDP infants received intervention for hypoglycaemia alone (i.v. dextrose) with no other major intervention (respiratory support, parenteral nutrition, central line, arterial line or blood transfusion).

**Conclusions:**

The burden of maternal HDP on late preterm and term admissions to NNU is high, with hypoglycaemia and respiratory disease being the main drivers for admission. Over one in four were admitted solely for management of hypoglycaemia. Further research should determine whether maternal antihypertensive agent choice or postnatal pathways may reduce NNU admission.

## Abstract 2

This article includes Author Insights, a video abstract available at: https://youtu.be/WduDjFDGUTo.

## Graphical abstract

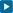

## 1 INTRODUCTION

Infant admission to a neonatal unit (NNU) often leads to separation of mother and baby during a critical period of bonding.^1^ Previous studies have estimated that one in five term admissions to NNU could be avoided.^2^ The most common primary reasons for term admissions to NNU are respiratory disease (~24% of admissions), infection (~18% of admissions) and hypoglycaemia (~10% of admissions).^2^

Maternal hypertensive disorders are a common complication of pregnancy (affecting ~10% of pregnancies) and a leading cause of perinatal morbidity and mortality including stillbirth, preterm birth, low birthweight and admission to a NNU.^3^, ^4^, ^5^, ^6^, ^7^ Whereas the high perinatal risk of hypertensive disorders is well described, the burden presented on NNU at a population level in the UK, and opportunities to avoid NNU admission in this cohort are not well characterised.

In comparison with infants born to normotensive mothers, infants born to mothers with hypertensive disorders of pregnancy (HDP) have a higher risk of preterm birth,^5^, ^8^ with concomitant risks of prematurity including respiratory morbidity.^9^ HDP infants also have a higher risk of hypoglycaemia as a result of fetal growth restriction secondary to poor placental function and iatrogenic causes secondary to maternal antenatal antihypertensive agent use (although evidence of the neonatal effects of in utero antihypertensive agent exposure remains sparse and conflicting),^10^, ^11^, ^12^, ^13^, ^14^ and antenatal corticosteroid use in preterm birth.^15^, ^16^, ^17^ Whereas antenatal corticosteroid use to reduce respiratory morbidity in preterm infants is well established,^18^ strategies to reduce infant hypoglycaemia in the context of HDP are lacking.

As previous studies have demonstrated that almost 100% of infants born at 25–33 weeks’ gestation are admitted to NNU for management of prematurity,^19^ infants born at ≥34 weeks’ gestation were the focus of this study where there may be opportunities to avoid NNU admission. We aimed to quantify the contribution of maternal HDP on NNU at a population level and identify opportunities to avoid NNU admission among late preterm and term infants.

## 2 METHODS

This was a retrospective cohort study using prospective, deidentified, routinely recorded neonatal electronic health record data from the National Neonatal Research Database (NNRD, Research Ethics Service approval [10/H0803/151]). The study was prospectively registered (Clinical Trials registration: NCT05015049) and received ethical approval (21/ES/0061). Findings are reported in line with RECORD guidelines.^20^ Data items are limited to what is held in the NNRD, but outcomes have been chosen to align with the neonatal core outcome set where possible.^21^ Parents were not involved in the development of this study.

### 2.1 Data sources

The NNRD has complete coverage of infants admitted to National Health Service (NHS) NNU in England and Wales since 2012.^19^ High completeness and accuracy (>95%) of neonatal data held in the NNRD has been confirmed by formal comparison with data from a multi‐centre, randomised placebo‐controlled trial.^19^ Population‐level Office of National Statistics (ONS) England and Wales annual livebirth data by gestation were accessed for denominator data.^22^

### 2.2 Study population

Infants born between 1 January 2012 and 31 December 2020 at ≥34 weeks’ gestation admitted to an NHS NNU in England or Wales were eligible for inclusion in this study. Infants with a confirmed congenital abnormality, those with implausible birthweight *Z*‐scores (≤−4 or ≥4), those with unspecified location of death (NNU versus home or hospice), and those with missing final discharge destination were excluded. We defined late preterm infants as those born between 34^+0^ and 36^+6^ weeks’ gestation and term infants as those born at ≥37 weeks’ gestation.

### 2.3 Exposure

To identify infants of mothers with HDP, maternal HDP diagnoses were extracted from antenatal, delivery and neonatal NNRD data items (Table S1). A single, mutually exclusive diagnosis was assigned (Table S2), with aggregation of diagnoses into three groups with shared pathophysiology for analysis: (i) ‘gestational hypertension’ – gestational hypertension or HDP not otherwise specified (maternal antihypertensive agent exposure in labour), (ii) ‘pre‐eclampsia’ – pre‐eclampsia or superimposed pre‐eclampsia (pre‐eclampsia in women with chronic hypertension) and (iii) chronic hypertension.

### 2.4 Outcomes

The primary outcome was primary reason for admission (single reason permitted on electronic health record system) extracted from the infant's first NNU admission. Secondary outcomes were survival, clinical diagnoses (ascertained from diagnosis codes across total NNU stay) and resource use (ascertained from daily data across total NNU stay). Daily level of care was determined according to Health Resource Group (HRG) definitions^23^ based on category of care received.^24^ Major NNU interventions were defined as any form of respiratory support, parenteral nutrition, central line, arterial line or blood transfusion. Primary management of hypoglycaemia alone was defined as receipt of i.v. dextrose and no other major intervention on infants cared for on special care (SC) only. We used number of days of i.v. dextrose as a proxy for hypoglycaemia severity. As this was highly skewed (median 2 days, range 1–121 days), we categorised this to a binary outcome variable of <3 days and ≥3 days. Birthweight *Z*‐scores were calculated with UK90 reference data using the LMS2z function of the SITAR package.^25^ Derived variable extraction procedures are listed in Table S3.

### 2.5 Statistical methods

Population proportions are reported with 95% confidence intervals (CIs) calculated using the exact binomial test. Differences in characteristics, outcomes and resource use between HDP and non‐HDP infants are described, with use of unadjusted and adjusted linear and logistic regression models for continuous and binary outcomes, respectively, and chi‐square tests for formal testing of differences.

To assess opportunities to avoid admissions in HDP infants, the intersection of maternal HDP status with currently recognised risk factors for infant hypoglycaemia as specified in the 2017 British Association of Perinatal Medicine (BAPM) Infant Hypoglycaemia guidelines^26^ (maternal diabetes, birthweight <2nd centile, late preterm birth) is reported. In addition, unadjusted and adjusted logistic regression models were used to test the association between maternal HDP, recognised infant hypoglycaemia risk factors and severity of hypoglycaemia (≥3 days of i.v. dextrose administration).

For all models, highly skewed variables violating model assumptions were log‐transformed. Missing data are reported in descriptive tables. All data processing and analysis was performed in R version 4.1.3 (10 March 2022).^27^

## 3 RESULTS

### 3.1 Burden of HDP on NNU admissions

**FIGURE 1:**
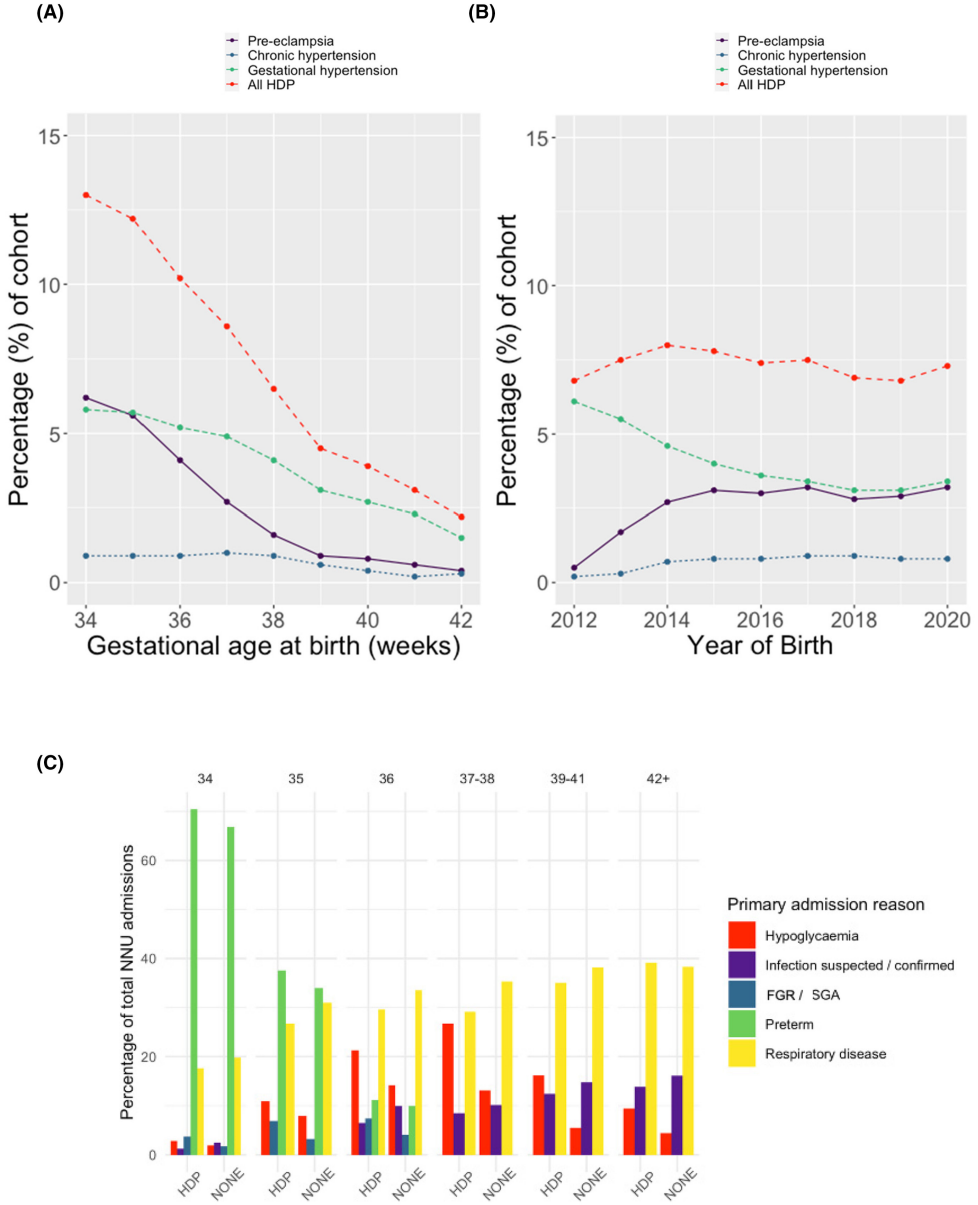
(A,B) Percentage of live‐born babies admitted to Neonatal Units in England and Wales 2012–2020 with a maternal record of hypertensive disorder of pregnancy by gestational age at delivery (A) and by year of birth (B). (C) Bar plot of proportion of infant admissions to a neonatal unit with primary diagnosis of prematurity, respiratory disease, hypoglycaemia, infection and growth restriction by gestational age (late preterm [34^+0^ to 36^+6^] by gestational week, early term [37^+0^ to 38^+6^], term [39^+0^ to 41^+6^] and post‐term [≥42^+0^]) stratified by exposure to maternal HDP. FGR, fetal growth restriction; HDP, hypertensive disorder of pregnancy; SGA, small for gestational age.

Of 5 950 223 recorded live births at ≥34 weeks’ gestation in England and Wales 2012–2020 (ONS), 420 866 infants (7.1% of all livebirths, 95% CI 7.1–7.1) meeting study inclusion criteria had an NNRD record of NNU admission (Figure S1). A maternal HDP was recorded in 30 944/420 866 of admitted infants (7.4%, 95% CI 7.3–7.4), with a higher burden in late preterm infants (16 059/136 220 [11.8%, 95% CI 11.6–12.0]) than term infants (14 885/284 646 [5.2%, 95% CI 5.2–5.3]; Figure 1A). Gestational hypertension was the most common recorded maternal HDP overall (Figure S1). Coding of gestational hypertension decreased and that of pre‐eclampsia increased between 2012 and 2016 (Figure 1B). This was consistent across all regions of England (Figure S2).

### 3.2 Characterisation of HDP infants

Infant antenatal and delivery characteristics are shown in Tables 1 and S4. HDP infants were born at earlier gestational ages than non‐HDP infants, with pre‐eclampsia infants being most likely to be born preterm (birth on average 12.9 days earlier than non‐HDP infants [95% CI 12.6–13.2 days]). Compared with non‐HDP infants, HDP infants also had higher odds of being small for gestational age (birthweight <10th centile), with the highest risk in the pre‐eclampsia subgroup (odds ratio [OR] 2.7, 95% CI 2.6–2.8). HDP infants were more likely to be born to women of black ethnic backgrounds in multivariable models adjusted for maternal age, parity and index of multiple deprivation compared with non HDP infants (aOR HDP infant black versus white ethnic background: 1.8, 95% CI 1.7–1.9, *P* < 0.0001).

**TABLE 1:** Baseline, antenatal and delivery characteristics of infants admitted to a neonatal unit at ≥34 weeks’ gestation in England and Wales 2012–2020 comparing those with and without a record of maternal hypertensive disorder of pregnancy (HDP) stratified by HDP type.

	Pre‐eclampsia (*n* = 10 821)	Chronic hypertension (*n* = 2959)	Gestational hypertension (*n* = 17 164)	All HDP (*n* = 30 944)	No HDP (*n* = 389 922)
Baseline infant characteristics					
Sex					
Female	4916 (45.43%)	1314 (44.41%)	7400 (43.11%)	13 630 (44.05%)	163 834 (42.02%)
Male	5897 (54.50%)	1644 (55.56%)	9755 (56.83%)	17 296 (55.89%)	225 811 (57.91%)
Missing	8 (0.07%)	1 (0.03%)	9 (0.05%)	18 (0.06%)	277 (0.07%)
Gestational age at delivery (weeks)	36.0 [34.9–37.4]	37.3 [35.7–39.0]	37.3 [35.6–39.1]	36.9 [35.14–38.6]	38.4 [36.3–40.1]
Birthweight (g)	2325 [1960–2910]	2900 [2320–3440]	2725 [2140–3370]	2580 [2070–3260]	3100 [2530–3600]
Missing	5 (0.05%)	1 (0.03%)	6 (0.03%)	12 (0.04%)	158 (0.04%)
Birthweight centile	32.8 (31.12)	46.3 (33.31)	38.9 (33.01)	37.5 (32.64)	46.9 (30.80)
Birthweight centile <10th	3605 (33.31%)	595 (20.11%)	4862 (28.33%)	9062 (29.29%)	60 411 (15.49%)
Number of fetuses					
Singleton	8941 (82.63%)	2716 (91.79%)	14 999 (87.39%)	26 656 (86.14%)	354 197 (90.84%)
Multi‐fetal	1878 (17.36%)	242 (8.18%)	2163 (12.60%)	4283 (13.84%)	35 575 (9.12%)
Missing	2 (0.02%)	1 (0.03%)	2 (0.01%)	5 (0.02%)	150 (0.04%)
Maternal age	31.0 [27.0–36.0]	34.0 [30.0–38.0]	31.0 [27.0–36.0]	32.0 [27.0–36.0]	31.0 [26.0–35.0]
Missing	148 (1.37%)	39 (1.32%)	221 (1.29%)	408 (1.32%)	8951 (2.30%)
Maternal parity					
Primiparous	5253 (48.54%)	847 (28.62%)	7262 (42.31%)	13 362 (43.18%)	128 822 (33.04%)
Missing	706 (6.52%)	141 (4.77%)	1794 (10.45%)	2641 (8.53%)	60 221 (15.44%)
Ethnicity (maternal)					
Asian	1064 (9.83%)	369 (12.47%)	1963 (11.44%)	3396 (10.97%)	38 405 (9.85%)
Black	760 (7.02%)	297 (10.04%)	1275 (7.43%)	2332 (7.54%)	16 550 (4.24%)
Mixed	129 (1.19%)	49 (1.66%)	187 (1.09%)	365 (1.18%)	4810 (1.23%)
Other	181 (1.67%)	47 (1.59%)	264 (1.54%)	492 (1.59%)	7591 (1.95%)
White	6517 (60.23%)	1645 (55.59%)	10 901 (63.51%)	19 063 (61.60%)	241 642 (61.97%)
Missing	2170 (20.05%)	552 (18.65%)	2574 (15.00%)	5296 (17.11%)	80 924 (20.75%)
Most deprived deprivation quintile (maternal)	2501 (23.11%)	936 (31.63%)	4447 (25.91%)	7884 (25.48%)	105 047 (26.94%)
Missing	321 (2.97%)	87 (2.94%)	524 (3.05%)	932 (3.01%)	14 374 (3.69%)
Antenatal variables					
Maternal medical history					
Diabetes	630 (5.82%)	471 (15.92%)	806 (4.70%)	1907 (6.16%)	10 294 (2.64%)
SLE	22 (0.20%)	14 (0.47%)	27 (0.16%)	63 (0.20%)	386 (0.10%)
Renal failure	34 (0.31%)	42 (1.42%)	21 (0.12%)	97 (0.31%)	175 (0.04%)
Renal transplant	9 (0.08%)	15 (0.51%)	9 (0.05%)	33 (0.11%)	59 (0.02%)
Obstetric complications					
Fetal growth restriction	1407 (13.00%)	285 (9.63%)	1935 (11.27%)	3627 (11.72%)	21 352 (5.48%)
Placental abruption	116 (1.07%)	25 (0.84%)	127 (0.74%)	268 (0.87%)	2116 (0.54%)
Gestational diabetes	1040 (9.61%)	419 (14.16%)	1987 (11.58%)	3446 (11.14%)	24 957 (6.40%)
Delivery characteristics					
Antenatal corticosteroids					
Complete	4619 (42.69%)	803 (27.14%)	4714 (27.46%)	10 136 (32.76%)	63 993 (16.41%)
Incomplete	511 (4.72%)	93 (3.14%)	477 (2.78%)	1081 (3.49%)	9836 (2.52%)
None given	2208 (20.40%)	794 (26.83%)	6773 (39.46%)	9775 (31.59%)	151 132 (38.76%)
Missing	3483 (32.19%)	1269 (42.89%)	5200 (30.30%)	9952 (32.16%)	164 961 (42.31%)
Onset of labour					
Not in labour	4640 (42.88%)	983 (33.22%)	4916 (28.64%)	10 539 (34.06%)	72 513 (18.60%)
Spontaneous	1496 (13.82%)	801 (27.07%)	4536 (26.43%)	6833 (22.08%)	184 402 (47.29%)
Induced	4051 (37.44%)	937 (31.67%)	6694 (39.00%)	11 682 (37.75%)	87 623 (22.47%)
Missing	634 (5.86%)	238 (8.04%)	1018 (5.93%)	1890 (6.11%)	45 384 (11.64%)
Mode of delivery					
Spontaneous vaginal delivery	1859 (17.17%)	787 (26.60%)	4446 (25.90%)	7092 (22.92%)	140 930 (36.14%)
Instrumental delivery	838 (7.65%)	243 (8.21%)	1868 (10.88%)	2939 (9.50%)	48 116 (12.34%)
Elective caesarean section	98 (0.90%)	35 (1.18%)	203 (1.18%)	336 (1.09%)	4328 (1.11%)
Emergency caesarean section	6176 (57.07%)	1169 (39.51%)	7732 (45.05%)	15 077 (48.72%)	107 352 (27.53%)
Missing	1860 (17.19%)	725 (24.50%)	2915 (16.98%)	5500 (17.78%)	89 196 (22.88%)

*Note*: Data are summarised as counts (%) for categorical data, mean (standard deviation) for approximately normally distributed continuous variables and median [interquartile range] for skewed continuous variables.

Abbreviation: SLE, systemic lupus erythematosus.

A total of 71.8% (95% CI 7.1–7.2) of HDP infants were born following obstetric intervention (pre‐labour caesarean section or induction of labour) compared with 41.1% (95% CI 41.0–41.2) of non‐HDP infants, with the highest proportion in the pre‐eclampsia subgroup (80.3%, 95% CI 79.6–81.1). HDP infants also had a high rate of emergency caesarean section (which includes expedited but non‐urgent caesarean sections) overall, with the highest rate in the pre‐eclampsia subgroup (Table 1).

### 3.3 Primary reasons for admission in HDP infants

Primary recorded reasons for admission are illustrated in Figure 1C and listed in Tables S5 and S6. The most common primary reason for admission in HDP infants overall was respiratory disease (27.8%, 95% CI 27.3–28.3), followed by prematurity (22.3%, 95% CI 21.8–22.8) and hypoglycaemia (16.1%, 95% CI 15.7–16.5). The majority of infants born from 34^+0^ to 35^+6^ weeks’ gestation were admitted with prematurity. From 36^+0^ to 41^+6^ weeks’ gestation, respiratory disease and hypoglycaemia were the most common reasons for admission. Infants in the gestational hypertension and pre‐eclampsia subgroups had the highest proportion of hypoglycaemia and prematurity admissions, respectively (Table S6).

Odds of admission with primary hypoglycaemia were twice as high in HDP as in non‐HDP infants across gestational age categories (overall OR 2.1, 95% CI 2.0–2.2, *P* < 0.0001), with the highest odds in the gestational hypertension subgroup (OR 2.5, 95% CI 2.4–2.6, *P* < 0.0001). A lower proportion of HDP compared with non‐HDP infants were admitted with respiratory disease and infection.

### 3.4 Neonatal outcomes in HDP infants

Hypertensive disorder of pregnancy infants had high survival rates (>99.5%) and these were higher than the rates for non‐HDP infants (OR 1.8, 95% CI 1.5–2.3, *P* < 0.0001). This was consistent across all HDP subtypes (Table 2). Compared with non‐HDP infants, HDP infants were more likely to have a record of hypoglycaemia during their NNU stay (overall OR 2.4, 95% CI 2.4–2.5, *P* < 0.0001) and diagnosis of growth restriction (overall OR 2.6, 95% CI 2.5–2.7, *P* < 0.0001). The highest odds of hypoglycaemia were observed in the gestational hypertension subgroup (OR 2.5 95% CI 2.4–2.6, *P* < 0.0001). The highest odds of growth restriction were observed in the pre‐eclampsia subgroup (OR 3.1, 95% CI 3.0–3.3, *P* < 0.0001).

**TABLE 2:** Neonatal outcomes and resource use of infants admitted to a neonatal unit at ≥34 weeks’ gestation in England and Wales 2012–2020 comparing those with and without a record of maternal hypertensive disorder of pregnancy (HDP) stratified by HDP type.

	Pre‐eclampsia (*n* = 10 821)	Chronic hypertension (*n* = 2959)	Gestational hypertension (*n* = 17 164)	All HDP (*n* = 30 944)	No HDP (*n* = 389 922)
NNU outcomes and diagnoses					
Survival to discharge					
Died	25 (0.23%)	14 (0.47%)	49 (0.29%)	88 (0.28%)	2040 (0.52%)
Growth restriction	2373 (21.93%)	408 (13.79%)	3075 (17.92%)	5856 (18.92%)	32 153 (8.25%)
Hypoglycaemia	4038 (37.32%)	1022 (34.54%)	6554 (38.18%)	11 614 (37.53%)	77 253 (19.81%)
Jaundice	3430 (31.70%)	881 (29.77%)	4560 (26.57%)	8871 (28.67%)	85 950 (22.04%)
Severe brain injury	264 (2.44%)	121 (4.09%)	585 (3.41%)	970 (3.13%)	16 465 (4.22%)
Seizures	138 (1.28%)	67 (2.26%)	331 (1.93%)	536 (1.73%)	8566 (2.20%)
Late onset sepsis	12 (0.11%)	2 (0.07%)	21 (0.12%)	35 (0.11%)	378 (0.10%)
NNU resource use					
Total length of stay	8 [4–14]	6 [3–11]	6 [3–11]	6 [3–12]	5 [3–9]
Highest level of care					
IC	1464 (13.53%)	505 (17.07%)	2204 (12.84%)	4173 (13.49%)	53 873 (13.82%)
HD	2583 (23.87%)	691 (23.35%)	3352 (19.53%)	6626 (21.41%)	84 620 (21.70%)
SC	6082 (56.21%)	1508 (50.96%)	9919 (57.79%)	17 509 (56.58%)	197 797 (50.73%)
Missing	692 (6.39%)	255 (8.62%)	1689 (9.84%)	2636 (8.52%)	53 632 (13.75%)
I.v. dextrose	7018 (64.86%)	1989 (67.22%)	10 928 (63.67%)	19 935 (64.42%)	233 098 (60.3%)
Received mechanical ventilation	926 (8.56%)	331 (11.19%)	1367 (7.96%)	2624 (8.48%)	37 630 (9.65%)
Received non‐invasive ventilation	3834 (35.43%)	1148 (38.80%)	5282 (30.77%)	10 264 (33.17%)	140 367 (36.00%)
Received parenteral nutrition	669 (6.18%)	159 (5.37%)	935 (5.45%)	1763 (6.70%)	16 317 (4.18%)
Blood products transfused	244 (2.25%)	95 (3.21%)	473 (2.76%)	812 (2.62%)	10 807 (2.77%)
Central venous line	1052 (9.72%)	354 (11.96%)	1636 (9.53%)	3042 (9.83%)	35 863 (9.2)
Umbilical arterial line	318 (2.94%)	157 (5.31%)	597 (3.48%)	1072 (3.46%)	17 341 (4.45%)
Peripheral arterial line	172 (1.59%)	74 (2.50%)	317 (1.85%)	563 (1.82%)	8117 (2.08%)
NG/NJ/OG feeding	8180 (77.2%)	1971 (68.4%)	11 653 (70.1%)	21 804 (70.46%)	219 332 (59.4%)
Peripheral venous line	8715 (80.54%)	2520 (85.16%)	13 989 (81.50%)	25 224 (81.52%)	322 522 (82.71%)

*Note*: Data are summarised as counts (%) for categorical data, mean (standard deviation) for approximately normally distributed continuous variables or median [interquartile range] for skewed continuous variables.

Abbreviations: HD, high dependency care; IC, intensive care; NG, naso‐gastric; NJ, naso‐jejunal; OG, oro‐gastric; SC, special care.

### 3.5 Resource use in HDP infants

Resource use is shown in Table 2. Total length of NNU stay was longer for all HDP subtypes compared with non‐HDP infants, and was highest in the pre‐eclampsia subgroup in keeping with earlier gestational age at delivery (43.4% longer, 95% CI 41.1–45.7, equivalent +2.6 days). The majority of HDP infants (17 509/30 944 [56.7%]) received SC only, with the highest proportion in the gestational hypertension subgroup (Table 2). The most common major interventions received by HDP infants were i.v. dextrose (64.5% of HDP infants overall versus 60.3% non‐HDP, *P* < 0.0001) and mechanical or non‐invasive ventilation (35.7% received versus 38.9% non‐HDP, *P* < 0.0001).

Of the HDP infants who received HD or IC care (*n* = 10 799), 8952 (81.2%) received mechanical or non‐invasive ventilation, 9900 (91.7%) received i.v. dextrose and 1746 (16.2%) received parenteral nutrition. These admissions were assessed as unlikely to be avoidable. Of HDP infants who received SC only (*n* = 17 509), a minority received non‐invasive ventilation (*n* = 1924 [11.1%]), 24 (0.1%) had a record of mechanical ventilation, 9780 (55.9%) received i.v. dextrose and none received parenteral nutrition.

In all, 8260/30 944 (26.7%) HDP infants received SC level care and intervention for hypoglycaemia (i.v. dextrose) and no other major intervention, suggesting admission primarily for management of hypoglycaemia alone. Gestational hypertension was the most common HDP subtype in these infants (4783/8260 [57.9%]) and median gestational age was 36.9 weeks’ gestation [interquartile range [IQR] 35.3–38.4].

In unadjusted analyses of the whole study cohort (*n* = 420 866), maternal HDP diagnosis was associated with primary management of hypoglycaemia alone (OR 1.4, 95% CI 1.3–1.4, *P* < 0.0001), with the highest odds in the gestational hypertension subgroup (gestational hypertension: OR 1.5, 95% CI 1.4–1.5, *P* < 0.0001, pre‐eclampsia: OR 1.3, 95% CI 1.2–1.4, *P* < 0.0001, chronic hypertension: OR 1.2, 95% CI 1.1–1.3, *P* = 0.0001). These associations remained strongly statistically significant overall (OR 1.3, 95% CI 1.2–1.3, *P* < 0.0001) and for gestational hypertension and pre‐eclampsia subgroups following adjustment for maternal diabetes status, birthweight centile, gestational age at delivery and fetal growth restriction diagnosis (gestational hypertension: OR 1.4, 95% CI 1.3–1.4, *P* < 0.0001, pre‐eclampsia: OR 1.2, 95% CI 1.1–1.2, *P* < 0.0001, chronic hypertension: OR 1.1, 95% CI 1.0–1.2, *P* = 0.0853).

### 3.6 Opportunities to avoid admission in HDP infants

**FIGURE 2:**
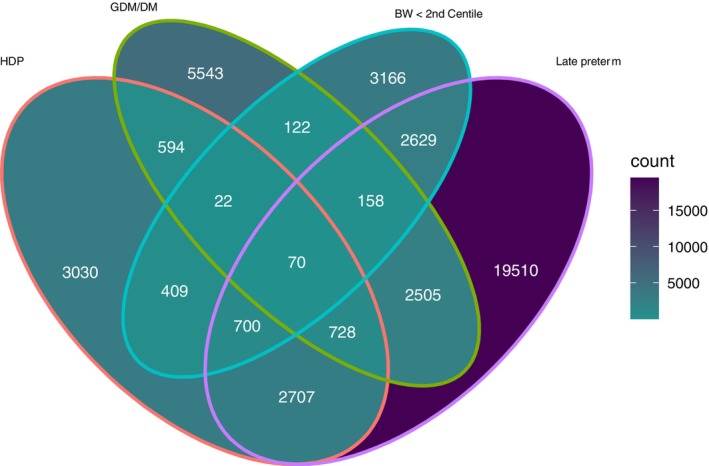
Venn diagram of recognised hypoglycaemia risk factors as defined in British Association of Perinatal Medicine 2017 Guidelines on Hypoglycaemia in term infant risk factors: maternal diabetes (DM, diabetes mellitus [Type 1 or 2]; GDM, gestational diabetes mellitus), birthweight [BW] <2nd centile and late preterm birth [34^+0^ to 36^+6^ weeks], in addition to hypertensive disorder of pregnancy [HDP] in late preterm and term infants admitted to NNU in England and Wales 2012–2020 who received primary management of hypoglycaemia alone (special care [SC] level care only, i.v. dextrose and no other major intervention). Total *n* = 89 381.

A Venn diagram of maternal HDP status and recognised neonatal hypoglycaemia risk factors^26^ in infants who received primary management for hypoglycaemia alone in the whole study population (*n* = 89 381) is shown in Figure 2. Of 8260 HDP infants in this group, the majority had HDP alone (*n* = 3030/8260 [36.7%]) or were born late preterm with HDP (2707/8260 [32.8%]) and 2523/8717 (30.5%) had multi‐factorial recognised risks for hypoglycaemia including maternal diabetes and/or birthweight <2nd centile. In sensitivity analysis using a birthweight centile threshold <10 and including antenatal steroid exposure where recorded, 2330/8260 (28.2%) of HDP infants had HDP alone, 702/8260 (8.5%) were additionally born late preterm and 996/8260 (12.1%) were additionally late preterm and had antenatal steroid exposure.

In adjusted logistic regression analysis of HDP infants receiving primary management for hypoglycaemia alone (*n* = 8260), birthweight <2nd centile and late preterm birth (34^+0^ to 36^+6^ weeks’ gestation) were the strongest predictors of i.v. dextrose administration for 3 days or more (Table S7). Furthermore, infants exposed to a maternal HDP with one or more recognised risk factors for hypoglycaemia (*n* = 5230, versus without additional risk factors, *n* = 3030) were substantially more likely to receive i.v. dextrose for ≥3 days (OR 1.7, 95% CI 1.5–1.8, *P* < 0.0001). The highest risk was among HDP infants with all three recognised risk factors (maternal diabetes, birthweight <2nd centile and late preterm birth, *n* = 70: OR 5.5, 95% CI 3.2–9.6, *P* < 0.0001; Table S8).

Overall, 3030/30 944 (9.8%, 95% CI 9.4–10.1) of HDP infants received primary management for hypoglycaemia with no additional recognised risk factors for infant hypoglycaemia. Gestational hypertension was the most common HDP diagnosis is this group of infants (2020/3030, 66.7%), followed by pre‐eclampsia (766/3030, 25.3%).

## 4 DISCUSSION

### 4.1 Main findings

Over one in ten late preterm and one in 20 term infants without congenital abnormalities admitted to NNU in England and Wales between 2012 and 2020 were associated with a recorded maternal HDP. Respiratory disease and hypoglycaemia were the primary pathologies driving HDP infant admission when considering both primary admission reason and interventions received. Overall, HDP infants were more than twice as likely than non‐HDP infants to be admitted with a primary reason of hypoglycaemia and have a hypoglycaemia diagnosis during admission.

We estimate approximately one in ten late preterm and term HDP infants admitted to NNU receive primary management of hypoglycaemia alone without additional recognised risk factors^26^ for infant hypoglycaemia. These infants were more likely to receive a short duration of i.v. dextrose (≤2 days). Infants born to mothers with gestational hypertension were the most likely to be admitted with a primary reason of hypoglycaemia, have a hypoglycaemia diagnosis, receive primary management of hypoglycaemia alone and have no additional risk factors. These findings suggest that interventions to reduce the risk and severity of infant hypoglycaemia in HDP could reduce neonatal unit admissions of late preterm and term infants of HDP mothers by at least 10%, especially in mothers with gestational hypertension. These infants may also be suitable for care in a transitional care setting without separation of mother and baby.

Compared with non‐HDP infants, infants in the pre‐eclampsia subgroup were the most likely to be born preterm, be delivered by emergency caesarean section, be of low birthweight centile and have a diagnosis of growth restriction. The finding of higher survival among HDP than non‐HDP infants is expected given the comparison group were sick infants requiring admission to NNU, in addition to the fact that delivery in HDP is often indicated for primarily maternal as opposed to fetal reasons.

### 4.2 Strengths and limitations

The strengths of this study include the population‐level coverage of NNU admissions enabling the national burden of HDP on NNU to be assessed and known high‐quality neonatal data within the NNRD.^19^ In addition, availability of maternal comorbidities and pregnancy complications in the NNRD enabled characterisation of HDP subtypes. Although there was variation in HDP subtype coding between 2012 and 2016, the characteristics of HDP infants described in this study are in keeping with anticipated phenotypes, which is reassuring and highlights the potential utility of maternal variables in the NNRD for studies at the intersection of obstetrics and neonatology.

The richness of the NNRD data extract enabled detailed characterisation of the study cohort and adjustment for relevant confounders in statistical models, such as demonstrating that HDP infants were more likely to receive primary management of hypoglycaemia alone after adjustment for maternal diabetes status, birthweight centile, gestational age at delivery and fetal growth restriction diagnosis. The variable ‘*primary reason for neonatal admission*’ reflected the admitting clinician's perception of the single most clinically significant issue, enabling us to highlight the burden of hypoglycaemia specifically in HDP infants. To mitigate the potential limitation of a single primary reason for admission, particularly prematurity, which can encompass multiple pathologies including respiratory disease and hypoglycaemia, we were able to use available daily resource data to confirm that i.v. dextrose and respiratory support were the most common major interventions late preterm and term HDP infants receive. The depth of information available also allowed us to examine days of i.v. dextrose as a proxy for hypoglycaemia severity, enabling further assessment of avoidability of hypoglycaemia admissions.

We were unable to examine effects of specific maternal antihypertensive agents, as these data are not captured routinely in the NNRD. Therefore third‐trimester in utero beta‐blocker exposure, which is another currently recognised risk factor for infant hypoglycaemia,^26^ could not be ascertained. As a result, we were unable to determine how many infants were exposed to labetalol and would have been additionally identified as high risk.

Maternal variables including HDP also may have been mis‐ or under‐ascertained, particularly because maternal diagnosis codes for missingness cannot be distinguished from absence of a condition. Efforts to provide robust national maternity data are ongoing.^28^ Should HDP infant live birth denominator data become available, linkage to the NNRD would enable interrogation of risk factors for NNU admission and hypoglycaemia in HDP. Formal validation of maternal variables in the NNRD with comparison with clinical codes and technical definitions, e.g. hypertension and proteinuria, would also be valuable.

### 4.3 Interpretation in the light of other studies

This is the first study, to our knowledge, of the population‐level contribution of HDP to late preterm and term infant NNU admissions in the UK and analysis of their outcomes, resource use and opportunities to avoid admission. International studies are limited. A population study of 6180 HDP infants born at 34^+0^ to 36^+6^ weeks’ gestation from the Netherlands also reported a higher risk of hypoglycaemia in infants born to women with pre‐eclampsia.^29^ A single‐centre UK study of 474 infants ≥35 weeks’ gestation reported maternal HDP were associated with infant hypoglycaemia admissions.^30^

### 4.4 Clinical relevance and future research

This study has highlighted that hypoglycaemia is a primary driver of late preterm and term HDP infant admissions in the UK. We estimate that over one in four admitted HDP infants receive management for hypoglycaemia alone, and one in ten additionally have no other recognised risk factors for hypoglycaemia, with gestational hypertension being the most common HDP subtype in these infants. It is likely that a high proportion of HDP infants in this study were exposed to a beta‐blocker in utero, given that current NICE Hypertension in pregnancy guidelines (CG107) recommend labetalol, a beta‐blocker, as first‐line treatment for pregnancy hypertension,^31^ primarily as labetalol has a licence for this use in pregnancy.

Maternal antihypertensive agent choice is a potentially modifiable risk factor for infant hypoglycaemia in HDP. Although epidemiological evidence strongly suggests beta‐blockers are associated with neonatal hypoglycaemia,^12^ a recent single‐centre study also reported nifedipine was associated with length of stay in infant hypoglycaemia.^30^ Clarification of the neonatal effects of antenatal and peri‐delivery exposure to antihypertensive agents is required. Should risk of neonatal hypoglycaemia vary substantially across antihypertensive agents, appropriate antenatal and peri‐delivery antihypertensive agent selection and postnatal risk stratification pathways may avoid at least 10% of current HDP NNU admissions.

### 4.5 Conclusion

This study demonstrates the high prevalence of maternal HDP in NNU admissions: one in ten late preterm and one in 20 term infant admissions to NNUs are associated with a maternal HDP. Respiratory disease and hypoglycaemia are the major drivers of admission in HDP infants. Examination of resource use data demonstrated that one in four HDP infants are being managed primarily for hypoglycaemia on SC. These admissions were assessed as being the most likely to be avoidable, particularly those infants with no additional risk factors for hypoglycaemia (one in ten HDP admissions overall). Our findings suggest that understanding the risk of hypoglycaemia across different maternal antihypertensive agents, appropriate postnatal pathways and consideration of transitional neonatal care, particularly in mothers with gestational hypertension, may substantially reduce the burden of maternal HDP on NNU and separation of mothers and their infants by reducing late preterm and term infant admissions for hypoglycaemia.

## AUTHOR CONTRIBUTIONS

FCR, LCC and CB conceived the study. JL accessed the NNRD database and performed data extraction. JL and JF performed data cleaning. FCR, JF and JL carried out data analysis. FCR drafted the first version of the paper, which was edited and approved by all the other authors.

## FUNDING INFORMATION

This work was supported by an Isaac Schapera Trust grant and the Medical Research Council (MR/V006835/1, external peer review). Professor Lucy Chappell is supported by an NIHR Senior Investigator Award. Dr Cheryl Battersby is funded by the UK NIHR through an Advanced Fellowship Award. The funders have played no role in conducting the research or writing the paper. For the purpose of open access, the author has applied a 'Creative Commons Attribution (CC–BY)' licence to any Author Accepted Manuscript (AAM) version arising.

## CONFLICT OF INTEREST STATEMENT

CB is funded by the UK NIHR through an Advanced Fellowship Award, has received support from Chiesi Pharmaceuticals to attend educational conferences, and been investigator on research grants from the National Institute of Health Research, CB is deputy chair for the NIHR Prioritisation committee for Hospital‐based care.

## ETHICS APPROVAL

The study was prospectively registered (Clinical Trials registration: NCT05015049) and received ethical approval from the HRA and Health and Care Research Wales (HCRW) committee on 29 June 2021 (REC reference: 21/ES/0061).

## Supporting information


Appendix S1.

## Data Availability

Data are available upon reasonable request. Applications to use the data presented within this project should be made to the Neonatal Data Analysis Unit, Imperial College London.
