# Supplementary material for: The contribution of hypertensive disorders of pregnancy to late preterm and term admissions to neonatal units in the UK 2012–2020 and opportunities to avoid admission: A population‐based study using the National Neonatal Research Database
Source: BJOG. 2023 Jun 19;131(1):88–98. doi: 10.1111/1471-0528.17574 (PMC10767760; doi:10.1111/1471-0528.17574)
Supplement: Supplementary file 1 — Appendix S1. [file BJO-131-88-s001.docx]

**Supplementary Tables**

**Table S1.** NNRD extraction procedures for hypertensive disorder of pregnancy diagnoses.

| **Variable** | **NNRD extraction procedure** |
| --- | --- |
| Chronic hypertension (CHT) | *Antenatal variables*  Variable: ProblemsMedicalMother  Coding: Dichotomous, code 12 (chronic hypertension) = TRUE, other codes = FALSE. |
| Gestational hypertension (GH) | *Antenatal variables*  Variable: *ProblemsDuringPregnancy*  Coding: Dichotomous, code 30 (Pregnancy induced hypertension) = TRUE, other codes = FALSE. |
| Pre-eclampsia (PET) | *Antenatal variables*  Variable: *ProblemsDuringPregnancy*  Coding: Code 31 (Pre-eclampsia) = TRUE  AND/OR  *Discharge diagnosis table*  Coding: TRUE if any of the following codes:  15389 (mild preeclampsia)  15390 (moderate preeclampsia)  15391 (severe preeclampsia) |
| Haemolysis, elevated liver-enzymes and low platelets syndrome (HELLP) | *Antenatal variables*  Variable: *ProblemsDuringPregnancy*  Coding: Dichotomous, code 32 (maternal HELLP) = TRUE, other codes = FALSE. |
| Hypertensive disorder not otherwise specified (NOS) | *Labour and delivery*  Variable: Drugs in Labour  Coding: Dichotomous, code 18 (antihypertensive) = 1, other codes = 0. |

**Table S2.** Hierarchical rules for assigning single, mutually exclusive hypertensive disorder of pregnancy diagnosis to infant.

| **Final hypertensive disorder of pregnancy diagnosis label** | **Definition (hierarchical)** |
| --- | --- |
| Superimposed pre-eclampsia | Chronic hypertension AND pre-eclampsia or HELLP with/without gestational hypertension with/without hypertensive disorder not otherwise specified |
| Chronic hypertension | Chronic hypertension with/without gestational hypertension with/without hypertensive disorder not otherwise specified |
| Pre-eclampsia | Pre-eclampsia or HELLP with/without gestational hypertension with/without hypertensive disorder not otherwise specified |
| Gestational hypertension | Gestational hypertension with/without hypertensive disorder not otherwise specified |
| Hypertensive disorder of pregnancy not otherwise specified | Hypertensive disorder of pregnancy not otherwise specified alone |

**Table S3.** NNRD extraction procedures and definition of derived outcomes and covariates.

| **Variable** | **NNRD extraction procedure** |
| --- | --- |
| Primary clinical diagnoses at first admission | Primary clinical diagnosis*  WHERE  Minimum admission time AND Episode Number = 1  *Coding:  10 - Preterm  11 - Respiratory disease  12 - Cardiovascular disease  13 - Failed oximetry testing  14 - Infection suspected / confirmed  15 - Jaundice  16 - Poor feeding or weight loss  17 - Weight loss  18 - Hypoglycaemia  19 - Convulsions suspected / confirmed  20 - Asphyxia  21 - Other neurological disease  22 - Congenital anomaly suspected / confirmed  23 - Social issues / Foster care  24 - Surgery  25 - Cardiac disease  26 - Investigation  27 - Monitoring (short observation)  28 - Poor condition at birth  29 - NAS suspected / confirmed  30 - HIE suspected / confirmed  31 - IUGR / SGA  32 - Birth trauma / injury  33 - Maternal admission / emergency  34 - Palliative care  35 - GIT disease  36 - Other metabolic disease  38 - Continuing care  39 - Re-admission  88 - Other  99 - Unknown |
| Survival | WHERE  Discharge destination at final admission = ‘Died’  THEN (0)  0 = Died  1 = Survived to discharge  9 = Unknown |
| Fetal growth restriction | *Antenatal variables*  Variable: *ProblemsDuringPregnancy*  Coding: TRUE if any of the following codes:  "Intrauterine growth restriction" (10)  "Poor biophysical profile" (11)  AND/OR  *Discharge diagnosis table*  Variables: *PrincipalDiagnosisAtDischarge* and text in diagnosis table have any of the following terms (== TRUE):  '15510': 'INTRAUTERINE GROWTH RESTRICTION (IUGR)'  '1010152': 'REVERSED DIASTOLIC FLOW (MATERNAL DOPPLERS)'  '15505': 'SMALL FOR DATES - NO MALNUTRITION (SGA/IUGR)'  '1010151': 'ABSENT DIASTOLIC FLOW (MATERNAL DOPPLERS)'  '15509': 'FETAL GROWTH RETARDATION'  '1010295': 'SMALL FOR GESTATIONAL AGE' |
| Hypoglycaemia diagnosis | The following terms or codes in discharge diagnoses or  the diagnosis table:  '15773': 'HYPERINSULINAEMIC HYPOGLYCAEMIA'  '15771': 'IATROGENIC NEONATAL HYPOGLYCAEMIA'/'HYPOGLYCAEMIA'/'NEONATAL HYPOGLYCAEMIA'/'OTHER NEONATAL HYPOGLYCAEMIA'  '500530': 'DRUG INDUCED HYPOGLYCAEMIA' |
| Jaundice | WHERE  Discharge diagnoses OR  Jaundice listed as primary clinical diagnosis for admission AND  Phototherapy given |
| Brain injury | For published definitions see:  Gale C, Statnikov Y, Jawad S On behalf of the Brain Injuries expert working group*, et al.* Neonatal brain injuries in England: population-based incidence derived from routinely recorded clinical data held in the National Neonatal Research Database. *Archives of Disease in Childhood - Fetal and Neonatal Edition*2018;**103:**F301-F306.   - Gale C, Ougham K, Jawad S, Uthaya S, Modi N. Brain injury occurring during or soon after birth: annual incidence and rates of brain injuries to monitor progress against the national maternity ambition 2018 and 2019 national data. *29*. 2021. doi:10.25561/87336   In term babies where:  ‘HIE’ = 1 OR  ‘IVH’ =1 OR  ‘PerinatalStroke’ =1 OR  ‘Kernicterus’ = 1 OR  ‘CNSInfection’ =1 OR  Seizures = 1  In preterm babies where:  ‘HIE’ = 1 OR  ‘IVH’ =1 OR  ‘PerinatalStroke’ =1 OR  ‘Kernicterus’ = 1 OR  ‘CNSInfection’ =1 OR  CPVL = 1 OR  Seizures = 1  THEN (1)  *Individual definitions:*  HIE: Defined as diagnoses of ‘Grade 3’ or ‘Grade 2’ HIE from all diagnosis fields in the NNRD  OR  Therapeutic Hypothermia for 2 or more consecutive days  IVH: Defined as diagnosis of ‘Grade ‘3’ or ‘Grade 4’ IVH from Cranial scans table in the NNRD  OR  Any of the following diagnoses in any of the diagnosis fields in the NNRD:   - SUBDURAL HAEMORRHAGE (DUE TO BIRTH INJURY) - CEREBRAL HAEMORRHAGE DUE TO BIRTH INJURY - TRAUMATIC INTRAVENTRICULAR HAEMORRHAGE - SUBARACHNOID HAEMORRHAGE - SUBARACHNOID HAEMORRHAGE DUE TO BIRTH INJURY - TENTORIAL TEAR DUE TO BIRTH INJURY - INTRACRANIAL LACERATION AND HAEMORRHAGE DUE TO BIRTH INJURY - INTRACRANIAL HAEMORRHAGE (UNKNOWN OR UNSPECIFIED CAUSE) - INTRACEREBRAL HAEMORRHAGE (TERM INFANT) - INTRAVENTRICULAR HAEMORRHAGE (PERINATAL) - POST-HAEMORRHAGIC HYDROCEPHALUS   OR  Any of the following procedures in any of the procedures fields in the NNRD:   - VENTRICULOPERITONEAL OR OTHER VENTRICULAR SHUNT - EXTERNAL VENTRICULAR DRAIN - VENTRICULAR DRAIN WITH RESERVOIR - INSERTION OF VENTRICULAR PERITONEAL SHUNT - INSERTION OF RICKHAM RESERVOIR - INSERTION OF VENTRICULO-ATRIAL CSF SHUNT - INSERTION OF VENTRICULO-PERITONEAL CSF SHUNT - CREATION OF VENTRICULOPERITONEAL SHUNT   PerinatalStroke: Defined as any of the following diagnoses in any of the diagnosis fields within the NNRD:   - NEONATAL STROKE - INFARCTION: MIDDLE CEREBRAL ARTERY - CEREBROVASCULAR ACCIDENT - CEREBRAL VENOUS THROMBOSIS - NEONATAL CEREBRAL ISCHAEMIA   Kernicterus:  Defined as any of the following diagnoses in any of the diagnosis fields within the NNRD:   - KERNICTERUS - BILIRUBIN ENCEPHALOPATHY   Seizures: Defined as any entry of ‘Convulsions’ in the Daily summary table in the NNRD  CNSinfection: Defined as any of the following diagnoses in any diagnosis field within the NNRD:   - ENCEPHALITIS - CANDIDA ENCEPHALITIS - BACTERIAL MENINGITIS - VIRAL MENINGITIS - MENINGITIS - BACTERIAL (SPECIFIC ORGANISM)   Specific options found in NNRD:   - - MENINGITIS – STREPTOCOCCAL   - MENINGITIS – CANDIDA - MENINGITIS - BACTERIAL (UNKNOWN OR UNSPECIFIED CAUSE) - CONGENITAL HERPES   OR  Any entry of any growth from a CSF culture  CPVL: Defined as being born at less than 37 weeks gestational age AND having any of the following diagnoses in any of the diagnosis field in the NNRD:   - CYSTIC PERIVENTRICULAR LEUKOMALACIA   OR  Being born < 37 weeks gestational age and having a diagnosis of PVL from Cranial scans table in the NNRD |
| Treated retinopathy of prematurity | WHERE  Surgery for ROP listed OR  Cryotherapy, laser therapy, anti-VEGF therapy, other treatment listed in right or left eye  OR  Avastin given  THEN == 1, ELSE == 0 |
| Bronchopulmonary dysplasia | WHERE  Any respiratory support given at WEEK 36 corrected gestational age  THEN (1)  0 = Died before week 36  1 = No respiratory support given at 36 weeks  2 = Respiratory support at 36 weeks  9 = Unknown  * Where no data for week 36 available due to early discharge, data is taken from the final or penultimate day on the unit |
| Severe necrotising enterocolitis | Necrotising enterocolitis resulting in death or requiring surgery  0 = No severe NEC  1 = Severe NEC present  9 = Unknown |
| Survival to discharge from neonatal care without comorbidity | Where  IF Treated_ROP = 0 AND BPD = 0 AND  SevereNEC = 0 AND Brain injury = 0 THEN 0  1 = Died or comorbidities present |
| Total length of stay in neonatal unit | SUM (Discharge time – Admission time) for each episode of care  In days |
| Number of days of intensive/high dependency/special care | SUM DAILY RECORDS WHERE  ‘HRG2016’ = intensive care / high dependency / special care as appropriate |
| Total days of mechanical ventilation (invasive respiratory support) | SUM DAILY SUMMARY RECORDS WHERE  Ventilation via endotracheal tube or tracheostomy  OR  Oxygen given with ventilation  OR  Conventional or high frequency oscillation |
| Total days of non-invasive respiratory support | SUM DAILY SUMMARY RECORDS WHERE  Non-invasive respiratory support (including CPAP)  OR  Oxygen given with CPAP, nasal ventilation, headbox oxygen, nasal cannula oxygen up to 1lpm, nasal cannula above 1lpm, high flow oxygen or air device used, or unspecified oxygen therapy  OR  Nasal CPAP (prong or mask), BIPAP/SIPAP, or high flow oxygen/air device given |
| Total days of IV dextrose | SUM DAILY SUMMARY RECORDS WHERE  IV Glucose electrolytes administered |

**Table S4.** Additional baseline, antenatal and delivery characteristics and resource use of infants admitted to a neonatal unit at ≥ 34 weeks gestation comparing those with and without a record of maternal hypertensive disorder of pregnancy (HDP) stratified by HDP type.

|  | | **Pre-eclampsia**  **N = 10,821** | **Chronic hypertension**  **N = 2,959** | **Gestational hypertension**  **N = 17,164** | **All HDP**  **N = 30,944** | **No HDP**  **N = 389,922** |
| --- | --- | --- | --- | --- | --- | --- |
|  | ***Baseline infant characteristics*** | | | | | |
| Gestational age category (weeks) | 34-36 | 7,200 (66.54%) | 1,253 (42.35%) | 7,606 (44.31%) | 16,059 (51.90%) | 120,161 (30.82%) |
|  | ≥37 | 3,621 (33.46%) | 1,706 (57.65%) | 9,558 (55.69%) | 14,885 (48.10%) | 269,761 (69.18%) |
| Birthweight centile <3rd |  | 1,775 (16.40%) | 278 (9.40%) | 2,458 (14.32%) | 4,511 (14.58%) | 26,783 (6.87%) |
| Geographic area | London | 1,849 (17.09%) | 576 (19.47%) | 3,417 (19.91%) | 5,842 (18.88%) | 64,795 (16.62%) |
|  | Midlands and East of England | 2,915 (26.94%) | 821 (27.75%) | 4,517 (26.32%) | 8,253 (26.67%) | 118,195 (30.31%) |
|  | North of England | 2,186 (20.20%) | 703 (23.76%) | 4,056 (23.63%) | 6,945 (22.44%) | 91,735 (23.53%) |
|  | South of England | 2,894 (26.74%) | 594 (20.07%) | 3,796 (22.12%) | 7,284 (23.54%) | 85,365 (21.89%) |
|  | Wales | 656 (6.06%) | 178 (6.02%) | 854 (4.98%) | 1,688 (5.46%) | 15,442 (3.96%) |
|  | *Missing* | 321 (2.97%) | 87 (2.94%) | 524 (3.05%) | 932 (3.01%) | 14,390 (3.69%) |
|  | ***Antenatal variables*** | | | | | |
| Maternal smoking in pregnancy |  | 868 (8.02%) | 400 (13.52%) | 1,621 (9.44%) | 2,889 (9.34%) | 53,336 (13.68%) |
|  | *Missing* | 1,020 (9.43%) | 265 (8.96%) | 1,701 (9.91%) | 2,986 (9.65%) | 66,338 (17.01%) |
|  | ***Delivery characteristics*** | | | | | |
| Maternal pyrexia in labour |  | 348 (3.22%) | 96 (3.24%) | 869 (5.06%) | 1,313 (4.24%) | 23,206 (5.95%) |
|  | *Missing* | 1,699 (15.70%) | 406 (13.72%) | 2,370 (13.81%) | 4,475 (14.46%) | 95,184 (24.41%) |
| Meconium stained liquor |  | 952 (8.80%) | 339 (11.46%) | 2,326 (13.55%) | 3,617 (11.69%) | 60,243 (15.45%) |
|  | *Missing* | 1,081 (9.99%) | 308 (10.41%) | 1,540 (8.97%) | 2,929 (9.47%) | 63,807 (16.36%) |
| Intrapartum antibiotics |  | 1,378 (12.73%) | 379 (12.81%) | 2,508 (14.61%) | 4,265 (13.78%) | 58,520 (15.01%) |
|  | *Missing* | 2,088 (19.30%) | 736 (24.87%) | 2,932 (17.08%) | 5,756 (18.60%) | 103,091 (26.44%) |
| Presentation at delivery | Cephalic | 8,110 (74.95%) | 2,182 (73.74%) | 13,437 (78.29%) | 23,729 (76.68%) | 287,767 (73.80%) |
|  | Breech | 985 (9.10%) | 233 (7.87%) | 1,279 (7.45%) | 2,497 (8.07%) | 23,552 (6.04%) |
|  | Transverse | 125 (1.16%) | 39 (1.32%) | 185 (1.08%) | 349 (1.13%) | 3,124 (0.80%) |
|  | *Missing* | 1,601 (14.80%) | 505 (17.07%) | 2,263 (13.18%) | 4,369 (14.12%) | 75,479 (19.36%) |
| 5min Apgar | Apgar < 7 | 1,005 (9.29%) | 290 (9.80%) | 1,505 (8.77%) | 2,800 (9.05%) | 34,562 (8.86%) |
|  | *Missing* | 543 (5.02%) | 154 (5.20%) | 976 (5.69%) | 1,673 (5.41%) | 45,350 (11.63%) |
|  | ***NNU outcomes*** | | | | | |
| Survival to discharge including comorbidity | Died | 25 (0.23%) | 14 (0.47%) | 49 (0.29%) | 88 (0.28%) | 2,040 (0.52%) |
|  | Survived to discharge with comorbidity | 249 (2.30%) | 113 (3.82%) | 562 (3.27%) | 924 (2.99%) | 15,322 (3.93%) |
|  | Survived to discharge without comorbidity | 10,542 (97.42%) | 2,831 (95.67%) | 16,548 (96.41%) | 29,921 (96.69%) | 372,428 (95.51%) |
|  | *Missing comorbidity status* | 5 (0.05%) | 1 (0.03%) | 5 (0.03%) | 11 (0.04%) | 132 (0.03%) |
|  | ***NNU resource use*** | | | | | |
| Length of IC-level care (days) |  | 6 [2-12] | 5 [2-10] | 5 [2-10] | 6 [2-11] | 4 [2-9] |
| Length of HD-level care (days) |  | 4 [2-10] | 3 [2-9] | 3 [2-9] | 4 [2-9] | 2 [1-6] |
| Length of SC-level care (days) |  | 10 [3-21] | 6 [2-17] | 6 [2-16] | 8 [3-18] | 4 [2-12] |

*Data are summarised as counts (%) for categorical data, mean (standard deviation) for approximately normally distributed continuous variables or median [interquartile range] for skewed continuous variables.*

*NNU: Neonatal Unit, IC: Intensive care, HD: High dependency care, SC: Special care.*

**Table S5.** Primary diagnosis or reason for admission at admission to neonatal unit in late preterm and term infants stratified by gestational age (in weeks) at delivery. The top five coded reasons for admission are listed comparing infants with and without a record of maternal hypertensive disorder of pregnancy (HDP), with all other reasons combined into ‘All other’.

| **Primary diagnosis at admission to NNU** | | | |
| --- | --- | --- | --- |
|  | **HDP** | **Non HDP** | **Ratio HDP: Non HDP** |
| **34^+0^ to 34^+6^ n = 6,312 (HDP) / n = 42,227 (Non HDP)** | | | |
| Preterm | 4,443 (70.4) | 28,188 (66.8) | 1.1 |
| Respiratory disease | 1,111 (17.6) | 8,366 (19.8) | 0.9 |
| IUGR / SGA | 231 (3.7) | 726 (1.7) | 2.1 |
| Hypoglycaemia | 178 (2.8) | 811 (1.9) | 1.5 |
| Infection suspected / confirmed | 76 (1.2) | 1,060 (2.5) | 0.5 |
| All other | 273 (4.3) | 3,076 (7.3) | 0.6 |
| **35^+0^ to 35^+6^ n = 5,025 (HDP) / n = 36,145 (Non HDP)** | | | |
| Preterm | 1,887 (37.6) | 12,292 (34.0) | 1.1 |
| Respiratory disease | 1,341 (26.7) | 11,190 (31.0) | 0.9 |
| Hypoglycaemia | 550 (10.9) | 2,872 (7.9) | 1.4 |
| IUGR / SGA | 345 (6.9) | 1,166 (3.2) | 2.1 |
| Other | 188 (3.7) | 942 (2.6) | 1.4 |
| All other | 714 (14.2) | 7,683 (21.3) | 0.7 |
| **36^+0^ to 36^+6^ n = 4,722 (HDP) / n = 41,789 (Non HDP)** | | | |
| Respiratory disease | 1,396 (29.6) | 14,044 (33.6) | 0.9 |
| Hypoglycaemia | 1,007 (21.3) | 5,946 (14.2) | 1.5 |
| Preterm | 531 (11.2) | 4,123 (9.9) | 1.1 |
| IUGR / SGA | 351 (7.4) | 1,720 (4.1) | 1.8 |
| Infection suspected / confirmed | 308 (6.5) | 4,128 (9.9) | 0.7 |
| Al other | 1,129 (23.9) | 11,828 (28.3) | 0.8 |
| **37^+0^ to 37^+6^ n = 4,775 (HDP) / n = 51,012 (Non HDP)** | | | |
| Respiratory disease | 1,378 (28.9) | 17,939 (35.2) | 0.8 |
| Hypoglycaemia | 1,253 (26.2) | 6,512 (12.8) | 2.1 |
| Jaundice | 511 (10.7) | 6,969 (13.7) | 0.8 |
| Infection suspected / confirmed | 347 (7.3) | 4,753 (9.3) | 0.8 |
| Other | 266 (5.6) | 2,392 (4.7) | 1.2 |
| All other | 1,020 (21.4) | 12,447 (24.4) | 0.9 |
| **38^+0^ to 38^+6^ n = 3,245 (HDP) / n = 46,571 (Non HDP)** | | | |
| Respiratory disease | 956 (29.5) | 16,485 (35.4) | 0.8 |
| Hypoglycaemia | 888 (27.4) | 6,264 (13.5) | 2.0 |
| Infection suspected / confirmed | 333 (10.3) | 5,199 (11.2) | 0.9 |
| Other | 179 (5.5) | 2,600 (5.6) | 1.0 |
| Monitoring (short observation) | 136 (4.2) | 2,487 (5.3) | 0.8 |
| All other | 753 (23.2) | 13,536 (29.1) | 0.8 |
| **39^+0^ to 39^+6^ n = 2,705 (HDP) / n = 56,849 (Non HDP)** | | | |
| Respiratory disease | 867 (32.1) | 21,576 (38.0) | 0.8 |
| Hypoglycaemia | 542 (20.0) | 4,272 (7.5) | 2.7 |
| Infection suspected / confirmed | 327 (12.1) | 7,674 (13.5) | 0.9 |
| Monitoring (short observation) | 150 (5.5) | 3,140 (5.5) | 1.0 |
| Other | 137 (5.1) | 2,994 (5.3) | 1.0 |
| All other | 682 (25.2) | 17,193 (30.2) | 0.8 |
| **40^+0^ to 40^+6^ n = 2,519 (HDP) / n = 61,672 (Non HDP)** | | | |
| Respiratory disease | 926 (36.8) | 23,125 (37.5) | 1.0 |
| Hypoglycaemia | 385 (15.3) | 3,021 (4.9) | 3.1 |
| Infection suspected / confirmed | 294 (11.7) | 9,514 (15.4) | 0.8 |
| Other | 128 (5.1) | 3,696 (6.0) | 0.8 |
| Monitoring (short observation) | 112 (4.4) | 3,179 (5.2) | 0.9 |
| All other | 674 (26.8) | 19,137 (31.0) | 0.9 |
| **41^+0^ to 41^+6^ n = 1,460 (HDP) / n = 45,725 (Non HDP)** | | | |
| Respiratory disease | 552 (37.8) | 18,113 (39.6) | 1.0 |
| Infection suspected / confirmed | 211 (14.5) | 7,162 (15.7) | 0.9 |
| Hypoglycaemia | 158 (10.8) | 1,748 (3.8) | 2.8 |
| Other | 77 (5.3) | 2,727 (6.0) | 0.9 |
| *Missing* | 74 (5.1) | 2,820 (6.2) | 0.8 |
| All other | 388 (26.6) | 13,155 (28.8) | 0.9 |
| **42^+0^ or more n = 181 (HDP) / n = 7,932 (Non HDP)** | | | |
| Respiratory disease | 71 (39.2) | 3,035 (38.3) | 1.0 |
| Infection suspected / confirmed | 25 (13.8) | 1,278 (16.1) | 0.9 |
| Hypoglycaemia | 17 (9.4) | 346 (4.4) | 2.2 |
| Monitoring (short observation) | 14 (7.7) | 379 (4.8) | 1.6 |
| Poor feeding or weight loss | 9 (5.0) | 326 (4.1) | 1.2 |
| All other | 45 (24.9) | 2,568 (32.4) | 0.8 |

**Table S6.** Primary diagnosis or reason for admission at admission to neonatal unit in all late preterm and term infants stratified by HDP and HDP subtype. The top five coded reasons for admission are listed comparing infants with and without a record of maternal hypertensive disorder of pregnancy (HDP).

|  | **Pre-eclampsia**  **N = 10,821** | **Chronic hypertension**  **N = 2,959** | **Gestational hypertension**  **N = 17,164** | **All HDP**  **N = 30,944** | **No HDP**  **N = 389,922** |
| --- | --- | --- | --- | --- | --- |
| Preterm | 3,218 (29.7%) | 421 (14.2%) | 3,257 (19.0%) | 6,896 (22.3%) | 45,048 (11.6%) |
| Respiratory disease | 2,887 (26.7%) | 967 (32.7%) | 4,744 (27.6%) | 8,598 (27.8%) | 133,873 (34.3%) |
| IUGR / SGA | 570 (5.3%) | 114 (3.9%) | 457 (2.7%) | 1,141 (3.7%) | 5,854 (1.5%) |
| Hypoglycaemia | 1,428 (13.2%) | 437 (14.8%) | 3,113 (18.1%) | 4,978 (16.1%) | 31,792 (8.2%) |
| Infection suspected / confirmed | 697 (6.4%) | 272 (9.2%) | 1,122 (6.5%) | 2,091 (6.8%) | 42,925 (11.0%) |

**Table S7.** Multiple logistic regression analysis of recognised infant hypoglycaemia risk factors, hypertensive disorder of pregnancy subtype and number of days of IV dextrose administration (binary outcome: 3 days or more) in HDP infants receiving primary management of hypoglycaemia alone (n = 8260).

|  | **Odds ratio [95% confidence interval]** | **p-value** |
| --- | --- | --- |
| Hypertensive disorder of pregnancy diagnosis  *Chronic hypertension (reference)*  *Gestational hypertension*  *Pre-eclampsia* | -  0.92 [0.78-1.08]  1.05 [0.89-1.25] | -  0.2981  0.5626 |
| Birthweight < 2^nd^ centile | 2.16 [1.90-2.45] | **<0.0001** |
| Gestational diabetes | 1.04 [0.91-1.20] | 0.5397 |
| Diabetes mellitus (Type 1 or 2) | 1.07 [0.90-1.29] | 0.4283 |
| Gestational age (weeks)  *34-36^+6^*  *37-38^+6^*  *39-41^+6^*  *42+* | 1.62 [1.04-1.35]  1.18 [1.04-1.35]  -  1.25 [0.67-2.32] | **<0.0001**  0.0125  -  0.4902 |

**Table S8.** Multiple logistic regression analysis of number of recognised infant hypoglycaemia risk factors and number of days of IV dextrose administration (binary outcome: 3 days or more) in HDP infants receiving primary management of hypoglycaemia alone (n = 8260).

|  | **Odds ratio [95% confidence interval]** | **p-value** |
| --- | --- | --- |
| 0 | *Ref* | - |
| 1 | 1.45 [1.32-1.60] | **<0.0001** |
| 2 | 2.17 [1.91-2.46] | **<0.0001** |
| 3 | 5.52 [3.18-9.58] | **<0.0001** |

**Supplementary Figures**

**Figure S1.** Study flow diagram.

**
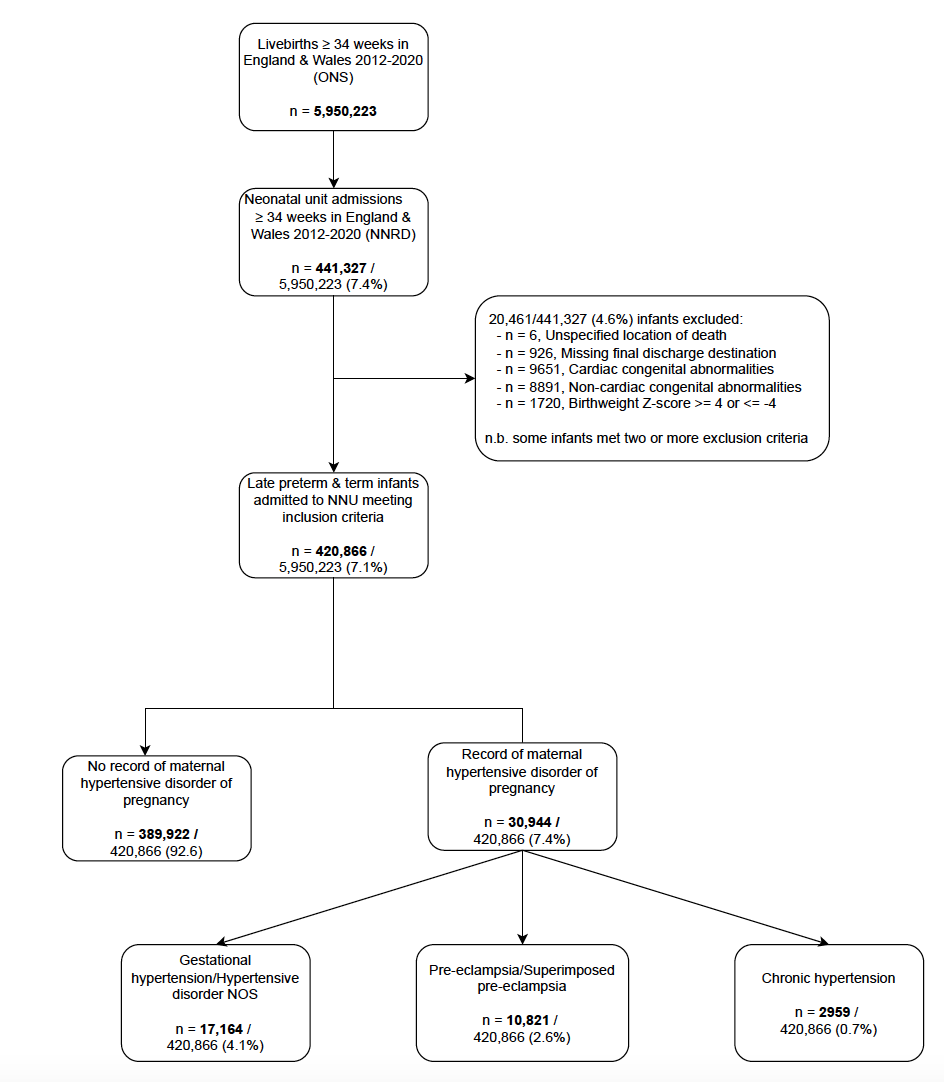
**

*ONS = Office of National Statistics, NNRD = National Neonatal Research Database, NOS = not otherwise specified.*

**Figure S2.** Proportion of infants born to mothers with a recorded diagnosis of A: pre-eclampsia, B: gestational hypertension, by year of birth and geographical region.


***
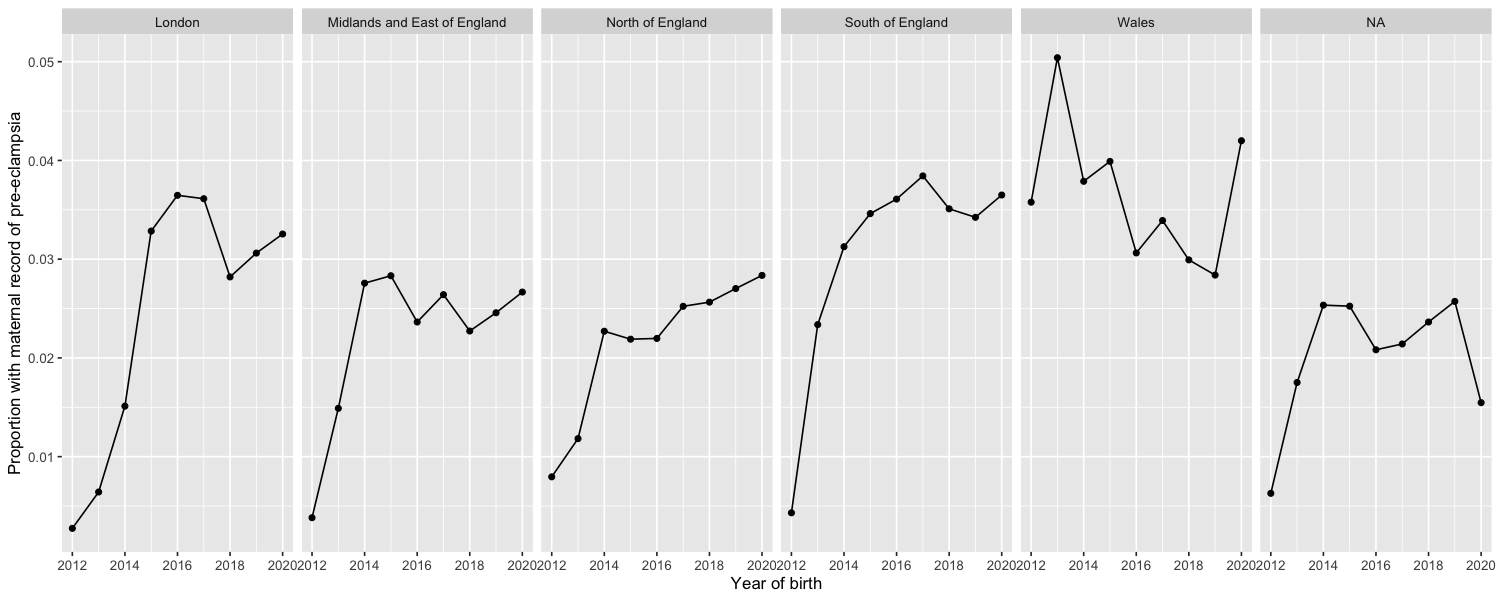
***


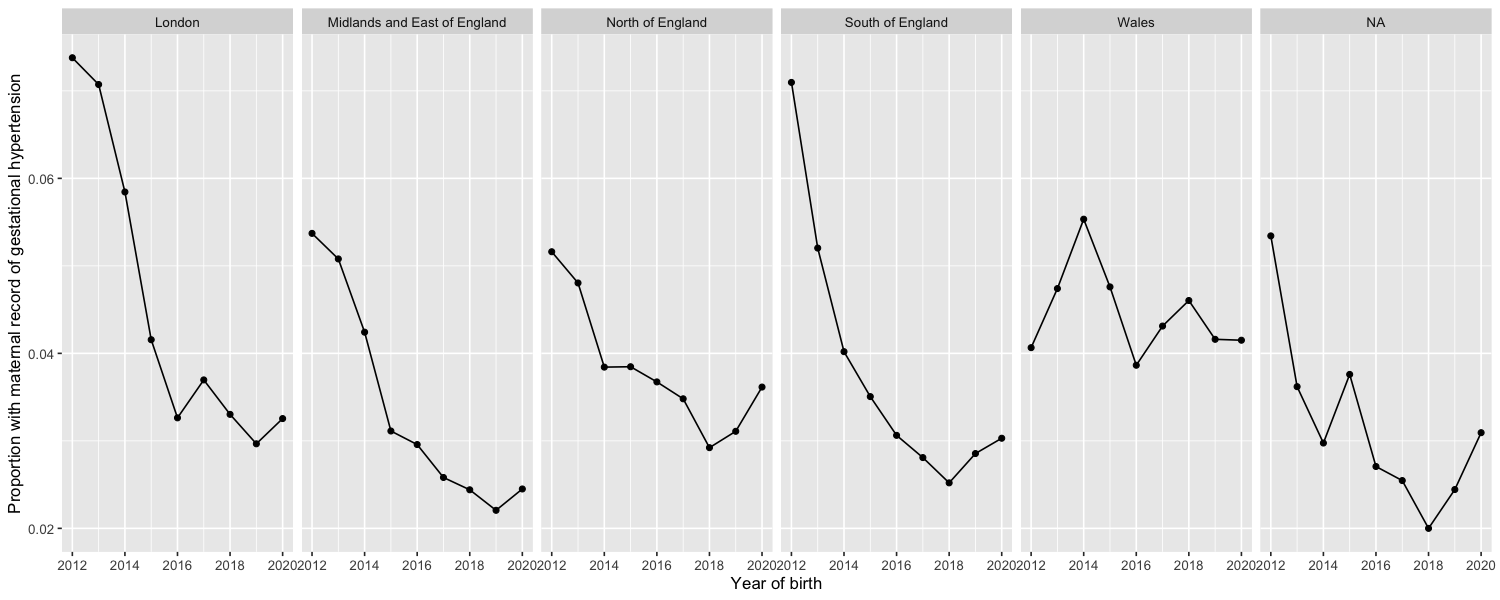


**Tables for Figure 2**

**Table S9.** Data for Figure 2A.

| **Birth Year** | **Hypertensive disorder of pregnancy type** | **Count** | **% total annual admissions** |
| --- | --- | --- | --- |
| 2012 | Pre-eclampsia | 249 | 0.5 |
| 2012 | Chronic hypertension | 69 | 0.2 |
| 2012 | Gestational hypertension | 2774 | 6.1 |
| 2013 | Pre-eclampsia | 778 | 1.7 |
| 2013 | Chronic hypertension | 159 | 0.3 |
| 2013 | Gestational hypertension | 2513 | 5.5 |
| 2014 | Pre-eclampsia | 1286 | 2.7 |
| 2014 | Chronic hypertension | 325 | 0.7 |
| 2014 | Gestational hypertension | 2214 | 4.6 |
| 2015 | Pre-eclampsia | 1539 | 3.1 |
| 2015 | Chronic hypertension | 392 | 0.8 |
| 2015 | Gestational hypertension | 1984 | 4 |
| 2016 | Pre-eclampsia | 1499 | 3 |
| 2016 | Chronic hypertension | 424 | 0.8 |
| 2016 | Gestational hypertension | 1802 | 3.6 |
| 2017 | Pre-eclampsia | 1557 | 3.2 |
| 2017 | Chronic hypertension | 466 | 0.9 |
| 2017 | Gestational hypertension | 1679 | 3.4 |
| 2018 | Pre-eclampsia | 1326 | 2.8 |
| 2018 | Chronic hypertension | 436 | 0.9 |
| 2018 | Gestational hypertension | 1465 | 3.1 |
| 2019 | Pre-eclampsia | 1294 | 2.9 |
| 2019 | Chronic hypertension | 369 | 0.8 |
| 2019 | Gestational hypertension | 1353 | 3.1 |
| 2020 | Pre-eclampsia | 1293 | 3.2 |
| 2020 | Chronic hypertension | 319 | 0.8 |
| 2020 | Gestational hypertension | 1380 | 3.4 |
| 2012 | All HDP | 3092 | 6.8 |
| 2013 | All HDP | 3450 | 7.5 |
| 2014 | All HDP | 3825 | 8 |
| 2015 | All HDP | 3915 | 7.8 |
| 2016 | All HDP | 3725 | 7.4 |
| 2017 | All HDP | 3702 | 7.5 |
| 2018 | All HDP | 3227 | 6.9 |
| 2019 | All HDP | 3016 | 6.8 |
| 2020 | All HDP | 2992 | 7.3 |

**Table S10.** Data for Figure 2B.

| **Gestation in Weeks** | **Hypertensive disorder of pregnancy type** | **Count** | **% of admissions by gestational age** |
| --- | --- | --- | --- |
| 34 | Pre-eclampsia | 3019 | 6.2 |
| 34 | Chronic hypertension | 455 | 0.9 |
| 34 | Gestational hypertension | 2838 | 5.8 |
| 35 | Pre-eclampsia | 2294 | 5.6 |
| 35 | Chronic hypertension | 371 | 0.9 |
| 35 | Gestational hypertension | 2360 | 5.7 |
| 36 | Pre-eclampsia | 1887 | 4.1 |
| 36 | Chronic hypertension | 427 | 0.9 |
| 36 | Gestational hypertension | 2408 | 5.2 |
| 37 | Pre-eclampsia | 1496 | 2.7 |
| 37 | Chronic hypertension | 536 | 1 |
| 37 | Gestational hypertension | 2743 | 4.9 |
| 38 | Pre-eclampsia | 782 | 1.6 |
| 38 | Chronic hypertension | 424 | 0.9 |
| 38 | Gestational hypertension | 2039 | 4.1 |
| 39 | Pre-eclampsia | 531 | 0.9 |
| 39 | Chronic hypertension | 351 | 0.6 |
| 39 | Gestational hypertension | 1823 | 3.1 |
| 40 | Pre-eclampsia | 512 | 0.8 |
| 40 | Chronic hypertension | 256 | 0.4 |
| 40 | Gestational hypertension | 1751 | 2.7 |
| 41 | Pre-eclampsia | 270 | 0.6 |
| 41 | Chronic hypertension | 115 | 0.2 |
| 41 | Gestational hypertension | 1075 | 2.3 |
| 42 | Pre-eclampsia | 29 | 0.4 |
| 42 | Chronic hypertension | 24 | 0.3 |
| 42 | Gestational hypertension | 123 | 1.5 |
| 34 | All HDP | 6312 | 13 |
| 35 | All HDP | 5025 | 12.2 |
| 36 | All HDP | 4722 | 10.2 |
| 37 | All HDP | 4775 | 8.6 |
| 38 | All HDP | 3245 | 6.5 |
| 39 | All HDP | 2705 | 4.5 |
| 40 | All HDP | 2519 | 3.9 |
| 41 | All HDP | 1460 | 3.1 |
| 42 | All HDP | 176 | 2.2 |
